# Supplementary material for: The genome sequence of the Ornate Brigadier fly, Odontomyia ornata (Meigen, 1822)
Source: Wellcome Open Res. 2025 May 23;10:272. [Version 1] doi: 10.12688/wellcomeopenres.24273.1 (PMC12739374; doi:10.12688/wellcomeopenres.24273.1)
Supplement: Peer review history

# Reviewer report: Reviewer response for version 1

**DOI:** 10.21956/wellcomeopenres.26770.r141438

**Published:** 2025-12-30

**Authors:** Charles Plessy

**Affiliations:** 1 Okinawa Institute of Science and Technology, Okinawa, Japan

This data note follows the standard structure used in the Tree of Life gateway. All genome quality indicators appear satisfactory. The BUSCO scores fall within the expected range for assemblies of related fly species. As of today, the closest species with a publicly released chromosome-level assembly is *Odontomyia argentata* (GCA_965615855.1). However, it is evolutionarily distant (~85% nucleotide sequence identity based on my own computation), making genome alignments unsuitable for detecting potential inconsistencies.

 I am assigning an “approved” status to this review.

 Nevertheless, I would appreciate if the authors could comment on the signal strength of interactions between the Y chromosome and the rest of the genome. I would have expected this to be roughly half of the average, similar to the X chromosome, given that sex chromosomes are haploid. However, the plot does not seem to reflect this. Could this be due to the Y chromosome being so short that the resolution of the plot does not allow this feature to be visible?

Are sufficient details of methods and materials provided to allow replication by others?

Yes

Is the rationale for creating the dataset(s) clearly described?

Yes

Are the datasets clearly presented in a useable and accessible format?

Yes

Are the protocols appropriate and is the work technically sound?

Yes

Reviewer Expertise:

Whole-genome alignments

I confirm that I have read this submission and believe that I have an appropriate level of expertise to confirm that it is of an acceptable scientific standard.

# Reviewer report: Reviewer response for version 1

**DOI:** 10.21956/wellcomeopenres.26770.r141431

**Published:** 2025-12-24

**Authors:** Darren Obbard

**Affiliations:** 1 The University of Edinburgh, Edinburgh, Scotland, UK

This data note reports the sequencing and assembly of the genome of *Odontomyia ornata* as part of the “Darwin Tree of Life” programme. In common with other data notes from this research effort, the reporting is standardised and quite brief. As such, I have very few comments to make.

 The approach is state-of-the-art, the raw data appear to be of a suitably high quality, and the assembly methods are appropriate. The public availability of raw data and genome assembly are appropriate. The resulting genome is likely to be of very high quality, and I have no doubt that it will be of great value to any researchers working on soldier flies and other diptera, or on the comparative or evolutionary genomics of insects more generally. I particularly applaud the efforts to identify sex chromosomes, and to report useful summary statistics such as heterozygosity

 At this point I usually have a number of minor suggestions focused on broadening the referenced literature and biology – but on this occasion, I really do not think there is anything to add. It’s great.

 My one suggestion is that, in addition to the sequenced specimen itself, it would be nice to have an ‘in life’ photograph and/or perhaps photographs both sexes. CC-BY images seem to be available from Wikimedia Commons and iNaturalist.

Are sufficient details of methods and materials provided to allow replication by others?

Yes

Is the rationale for creating the dataset(s) clearly described?

Yes

Are the datasets clearly presented in a useable and accessible format?

Yes

Are the protocols appropriate and is the work technically sound?

Yes

Reviewer Expertise:

Evolutionary genetics of invertebrates and their pathogens

I confirm that I have read this submission and believe that I have an appropriate level of expertise to confirm that it is of an acceptable scientific standard.

# Reviewer report: Reviewer response for version 1

**DOI:** 10.21956/wellcomeopenres.26770.r124409

**Published:** 2025-08-19

**Authors:** Andrés López-Rubio

**Affiliations:** 1 Tecnológico de Antioquia, Medellín, Colombia

A great research paper, it will contribute to the knowledge of Stratiomyid. As mentioned before, this paper out cases a remarkable analysis workflow to produce a fully annotated genome of a Stratiomyid fly. Previous to this paper NCBI reports only *Odontomyia argentata* to be sequenced. Since all the results are available, it will improve the sequencing of more species in the family.

Are sufficient details of methods and materials provided to allow replication by others?

Yes

Is the rationale for creating the dataset(s) clearly described?

Yes

Are the datasets clearly presented in a useable and accessible format?

Yes

Are the protocols appropriate and is the work technically sound?

Yes

Reviewer Expertise:

Bioinformatics, phylogenetics

I confirm that I have read this submission and believe that I have an appropriate level of expertise to confirm that it is of an acceptable scientific standard.
